# Poster Session I - A32 NOD2 DRIVES REGENERATIVE FETAL-LIKE REPROGRAMMING IN THE INTESTINAL EPITHELIUM

**DOI:** 10.1093/jcag/gwaf042.032

**Published:** 2026-02-13

**Authors:** D Tsang, C Maisonneuve, A Ayyaz, E Foerster, M Nissan, L Baerg, D Trcka, B Ghoshal, B K Tsankov, G Bayer, M Denney, C Streutker, J Wrana, S Girardin, D J Philpott

**Affiliations:** Immunology, University of Toronto, Toronto, ON, Canada; Lunenfeld-Tanenbaum Research Institute, Toronto, ON, Canada; University of Calgary, Calgary, AB, Canada; Immunology, University of Toronto, Toronto, ON, Canada; Immunology, University of Toronto, Toronto, ON, Canada; Immunology, University of Toronto, Toronto, ON, Canada; Lunenfeld-Tanenbaum Research Institute, Toronto, ON, Canada; Lunenfeld-Tanenbaum Research Institute, Toronto, ON, Canada; Immunology, University of Toronto, Toronto, ON, Canada; Immunology, University of Toronto, Toronto, ON, Canada; Immunology, University of Toronto, Toronto, ON, Canada; Unity Health Toronto, Toronto, ON, Canada; Lunenfeld-Tanenbaum Research Institute, Toronto, ON, Canada; Immunology, University of Toronto, Toronto, ON, Canada; Immunology, University of Toronto, Toronto, ON, Canada

## Abstract

**Background:**

Inflammatory injury to the intestine triggers a reprogramming of the intestinal epithelium to a fetal-like state that drives rapid restoration of the epithelial barrier. Although the intestinal microbiota is a key modulator of inflammation, its role in influencing epithelial fetal-like stem cell reprogramming and consequent restitution remains unclear.

**Aims:**

We aim to identify how host-microbiota interactions regulate intestinal epithelial restitution following damage. Our hypothesis is that intestinal epithelial NOD2 signaling accelerates epithelial fetal-like stem cell reprogramming to promote tissue repair following intestinal injury.

**Methods:**

Mice were irradiated (IR, 12Gγ) to induce synchronous inflammatory injury to small intestine. Intestinal restitution kinetics were assessed transcriptionally (scRNA-Seq, qPCR), histologically, and by flow cytometry. *In vivo* observations were supported with *in vitro* small intestinal organoid models of inflammatory injury.

**Results:**

We demonstrated that the intestinal microbiota accelerated epithelial restitution through an amplified inflammation-associated repair response that promoted the emergence of fetal-like intestinal epithelial cells (IECs) expressing *Ly6a* and *Clu*. NOD2, a sensor of bacterial peptidoglycan, whose gene has mutations associated with CD, was expressed in fetal-like IECs following injury. In ileal organoids, NOD2 stimulation potentiated an inflammatory gene signature associated with interferon (IFN) signaling, which was concomitant with enterocyte recovery. Deficiency in NOD2 exacerbated epithelial apoptosis following IR injury, while epithelial-specific NOD2 signaling increased the emergence of fetal-like IECs and consequently, increased epithelial proliferation.

**Conclusions:**

These findings identify a critical role for NOD2-dependent microbial detection in regulating the expansion of fetal-like IECs following injury, which contributes to the protective effect of this microbial sensor in resolving intestinal inflammation.

**Funding Agencies:**

CIHR

